# Taking antithrombic therapy during emergency laparoscopic cholecystectomy for acute cholecystitis does not affect the postoperative outcomes: a propensity score matched study

**DOI:** 10.1186/s12893-022-01501-6

**Published:** 2022-02-05

**Authors:** Kentaro Oji, Yasunori Otowa, Yuta Yamazaki, Keisuke Arai, Yasuhiko Mii, Keitaro Kakinoki, Tetsu Nakamura, Daisuke Kuroda

**Affiliations:** 1 Department of Surgery, Kita-Harima Medical Center, 926-250 Ichiba-cho, Ono, Hyogo 675-1392 Japan; 2 Radiation Biology Branch, National Cancer Institute, 9000 Rockville Pike, Bethesda, Maryland 20892 USA

**Keywords:** Antithrombic therapy, Acute cholecystitis, Laparoscopic cholecystectomy

## Abstract

**Background:**

Continuing antithrombic therapy (ATT) during surgery increases the risk of bleeding. However, it is difficult to discontinue the ATT in emergency surgery. Therefore, safety of emergency laparoscopic cholecystectomy (LC) for acute cholecystitis (AC) is still unclear. We aimed to clarify the affect of ATT during emergency LC for AC.

**Methods:**

Patients with AC were classified into ATT group (n = 30) and non-ATT group (n = 120). Postoperative outcomes were compared after propensity score matching (n = 22).

**Results:**

Higher level of c-reactive protein level and shorter activated partial thromboplastin time (APTT) was observed in ATT group than in non-ATT group after matching. No significant difference was found between other patient characteristics and perioperative results. Blood loss over 100 mL was observed in 8 patients. Multivariate analyze showed that APTT was an independent risk factor for bleeding over 100 mL (*P* = 0.039), while ACT and APT was not.

**Conclusions:**

Taking ATT does not affect the blood loss or complications during emergency LC for AC. Controlling intraoperative bleeding is essential for a safe postoperative outcome.

## Background

Antithrombic therapy (ATT) is used to prevent primary and secondary thromboembolic complications after cardiovascular or cerebrovascular diseases and the use of ATT is more relevant in elder population [1, 2]. However, continuing ATT increases the risk of bleeding and discontinuing the ATT increases the risk of thrombosis during perioperation [3–6]. Several reports showed that it was safe to continue the ATT during perioperation in abdominal surgery [7–9]. But still, thromboembolic and bleeding risks should be considered whether to interrupt or continue the ATT in elected surgery. On the other hand, bleeding is more relevant in inflammatory phase and it is difficult to interrupt ATT in emergency surgery which increases the risk of bleeding [10].

Laparoscopic cholecystectomy (LC) is a standard treatment for cholecystitis and also for acute cholecystitis (AC). Previous studies showed that LC can be performed safely in patients with ATT in acute phase [11–14]. However, there was a significant difference in patient characteristics of those taking ATT and those without ATT or the age was relatively young. Generally, as the age raise, higher rate of comorbidity and uses of ATT is relevant [15]. Therefore, the safety of emergency LC for AC with ATT remains unclear. In this retrospective study, we aimed to clarify the affect of ATT during emergency LC for AC.

## Materials and methods

We performed a retrospective review of all patients who underwent emergency LC for AC at our institution from October 2013 to October 2019. Patients treated with laparotomy, or who had gallbladder drainage before surgery were excluded from the study. Patients were classified into one of three severity grades by the 2018 Tokyo Guidelines (TG18) [16].

LC was performed with standard four-port technique. Extra port was added if necessary. Pneumoperitoneum pressures were maintained at 10 mmHg. Ultrasonic coagulating shears was mainly used around the Calot’s triangle to prevent bleeding from small vessels. Soft coagulation was used to stop the bleeding and keep the surgical field dry. Mechanical compression using hemostatic agent was performed when it was difficult to control the bleeding only by soft coagulation. Ultrasonic coagulating shears and soft coagulation was used in all cases. Postoperative complications were graded according to the Clavien–Dindo classification, and cases with a classification over grade II were defined as having a postoperative complication [17].

### Statistical analysis

Statistical analysis was carried out using the EZR software which is a graphical user interface for R (R Foundation for Statistical Computing, Vienna, Austria, v. 4.0.3). The following covariates were included in the score matching: age, American Society of Anesthesiologists-Physical Status (ASA-PS), Charlson comorbidity Index (CCI), c-reactive protein (CRP), hemoglobin, TG18 grade, platelet, sex, and white blood cell. The relation between two variables was assessed using the Fisher exact test or Chi-squared test and Student’s T-test or Mann–Whitney U test. Variables with a *P* value < 0.1 in a univariate analysis were further evaluated in a multivariate analysis using the logistic regression model to assess the confounding variables. In all analyses, *P* < 0.05 was accepted as statistically significant.

## Results

Two hundred one patients were diagnosed as AC and underwent surgery during the period. Fifty one patients were excluded: 50 patients due to gallbladder drainage before surgery and 1 patient due to laparotomy. One hundred fifty patients were analyzed. Patient characteristics are listed in Table 1. Thirty patients took ATT and 17 patients (56.7%) were over 75 years old. Seven patients (23.3%) had anticoagulation therapy (ACT) and 25 patients (83.3%) had antiplatelet therapy (APT). Five patients (16.7%) had multiple dosage of APT or combination of ACT and APT. Before matching, there were significant difference between groups in ASA-PS, CCI, TG18 grade, and blood test results. There was a trend of elder patients and more male in ATT group than in non-ATT group. Significantly higher ASA-PS and CCI was observed in ages over 75 years old compared with age under 75 years old (*P* = 0.032 and *P* < 0.001, respectively). After matching, 22 patients had ATT. Among them, 9 patients (40.9%) were over 75 years old. Higher CRP level, longer prothrombin time-international normalized ratio (PT-INR), and shorter activated partial thromboplastin time (APTT) was observed in ATT group than in non-ATP group (*P* = 0.002, *P* = 0.001, and *P* < 0.001, respectively). There was no difference in ASA-PS and CCI between ages over 75 years old and under.

**Table 1:** Patient characteristics

	Before match			After match		
	ATT (n = 30)	Non-ATT (n = 120)	*P* value	ATT (n = 22)	Non-ATT (n = 22)	*P* value
Age	77 (44–90)	70 (25–100)	0.065	77 (59–90)	72 (51–92)	0.549
Sex (Male/Female)	23/7	70/50	0.092	15/7	15/7	1.000
BMI (kg/m^2^)	23.1 (3.9)	23.9 (3.8)	0.302	22.5 (3.4)	22.4 (3.1)	0.973
ASA-PS (1,2/3,4)	16/14	105/15	< 0.001	14/8	17/5	0.510
CCI	1 (0–6)	0 (0–4)	0.001	1 (0–6)	1 (0–4)	0.714
TG18 grade (1/2,3)	8/22	78/42	< 0.001	8/14	10/12	0.760
WBC (10^4^/µL)	1.5 (0.5)	1.3 (0.5)	0.039	1.3 (0.5)	1.5 (0.4)	0.489
Hb (g/dL)	12.7 (1.7)	13.6 (2.0)	0.028	12.7 (1.4)	12.7 (2.4)	0.970
Plt (10^4^/µL)	21.0 (7.5)	23.2 (8.1)	0.180	21.5 (8.1)	23.1 (7.1)	0.489
CRP (mg/dL)	14.8 (0.1–44.1)	4.0 (0.0–18.4)	0.004	15.8 (0.1–44.1)	2.4 (0.0–30.9)	0.022
Alb (g/dL)	3.4 (0.7)	3.7 (0.7)	0.007	3.4 (0.7)	3.7 (0.8)	0.190
Total bilirubin (mg/dL)	1.2 (0.2–5.4)	1.0 (0.2–16.8)	0.195	1.1 (0.2–5.4)	0.9 (0.4–10.3)	0.597
AST	25 (14–513)	24 (11–1243)	0.842	24 (14–201)	23 (11–390)	0.787
ALT	19 (6–515)	24 (6–866)	0.491	17 (6–114)	20 (6–866)	0.526
ALP	273 (155–1369)	258 (115–1477)	0.521	256 (155–627)	270 (121–1477)	0.771
γGTP (IU/L)	50 (9–450)	40 (12–572)	0.932	47 (9–128)	43 (14–572)	0.488
Cr	0.8 (0.4–7.3)	0.8 (0.3–7.1)	0.116	0.8 (0.5–7.3)	0.8 (0.5–7.1)	0.953
PT-INR	1.2 (1.0–2.0)	1.1 (0.9–1.4)	< 0.001	1.2 (1.0–1.6)	1.0 (0.9–1.3)	0.001
APTT (s)	41.1 (26.6–76.1)	33.5 (21.4–53.6)	< 0.001			
ACT (%)	7 (23.3)			4 (18.2)		
APT (%)	25 (83.3)			18 (81.8)		
Multiple dosage (%)^a^	5 (16.7)			2 (9/1)		
Duration until operation from onset	3 (1–10)	2 (1–30)	0.088	3 (1–10)	3 (1–10)	0.157

Mean (SD) or median (range)

ACT, anticoagulation therapy; APTT, activated partial thromboplastin time; ATT, antithrombic therapy; BMI, body mass index; ASA-PS, American Society of Anesthesiologists-Physical Status; CCI, Charlson Comorbidity Index: TG18, Tokyo guideline 2018; WBC, white blood cell; Hb, hemoglobin, Plt, platelet; CRP, c-reactive protein; Alb, albumin, γGTP, gamma glutamyl transpeptidase; PT-INR, prothrombin time-international normalized ratio

^a^Multiple dosage of APT or combination of ACT and APT

The perioperative results are listed in Table 2. Before matching, one patient was converted to laparotomy in the non-ATT group. There was a trend of longer operation time and higher blood loss in ATT group compared with non-ATT group, although the difference was not significant. There was no difference in complications. After matching, there still was a trend of longer operation time and more blood loss in ATT group compared with non-ATT group, although the difference was not significant. There was no difference in complications and hospital stays.

**Table 2:** Perioperative results

	Before match			After match		
	ATT (n = 30)	Non-ATT (n = 120)	*P* value	ATT (n = 22)	Non-ATT (n = 22)	*P* value
Open conversion (%)	0	1 (0.8)	1.000	0	0	1.000
Operative time (min)	130 (79–260)	119 (51–305)	0.107	128 (79–260)	116 (54–232)	0.205
Blood loss (mL)	17 (0–651)	10 (0–550)	0.179	13 (0–651)	10 (0–378)	0.179
Blood loss > 100 mL (%)	7 (23.3)	21 (17.5)	0.446	6 (27.3)	2 (9.1)	0.240
All postoperative complications (%)^a^	4 (13.3)	14 (11.7)	0.759	3 (13.6)	4 (18.2)	1.000
Bleeding (%)	0	0	NA	0	0	NA
Abdominal abscess (%)	1 (3.3)	2 (1.7)	0.491	1 (4.5)	1 (4.5)	1.000
Bile leak (%)	0	4 (3.3)	0.584	0	0	NA
Respiratory (%)	1 (3.3)	2 (1.7)	0.491	1 (4.5)	0	1.000
Others (%)	1 (3.3)	4 (3.3)	1.000	0	2 (9.1)	0.488
Hospital stays (days)	10.5 (5–42)	7 (3–36)	0.002	11 (5–42)	7 (3–30)	0.409

Mean (SD) or median (range)

^a^Some cases overlapped

We further analyzed what affected the blood loss over 100 mL. The difference in patient characteristics according to the blood loss is listed in Table 3. There was a trend of lower albumin level and longer PT-INR and APTT in patients with blood loss over 100 mL compared with blood loss under 100 mL. Multivariate analyze (Table 4) showed that APTT is an independent risk factors for bleeding over 100 mL (*P* = 0.039), while ACT and APT was not.

**Table 3:** Patient characteristics according to the blood loss

	Blood loss ≥ 100 mL (n = 8)	Blood loss < 100 mL (n = 36)	*P* value
Age	79.6 (5.6)	72.7 (11.9)	0.119
Sex (Male/Female)	6/2	24/12	1.000
BMI (kg/m^2^)	22.1 (3.8)	22.5 (3.2)	0.719
ASA-PS (1,2/3,4)	5/3	26/10	0.676
CCI	1 (1–2)	1 (0–2)	0.699
TG18 grade (1/2,3)	2/6	16/20	0.439
WBC (10^4^/µL)	1.5 (0.7)	1.4 (0.4)	0.496
Hb (g/dL)	11.9 (1.7)	12.9 (2.0)	0.184
Plt (10^4^/µL)	20.3 (5.5)	22.8 (7.9)	0.416
CRP (mg/dL)	16.7 (6.3–27.6)	9.6 (0.0–44.1)	0.235
Alb (g/dL)	3.1 (0.5)	3.6 (0.7)	0.089
Total bilirubin (mg/dL)	1.2 (0.6–5.4)	0.9 (0.2–10.3)	0.429
AST	22 (14–62)	23 (11–390)	0.626
ALT	14 (7–47)	19 (6–866)	0.201
ALP	263 (202–627)	270 (121–1477)	0.629
γGTP (IU/L)	34 (9–87)	46 (14–572)	0.543
Cr	0.8 (0.5–4.2)	0.9 (0.5–7.3)	0.692
PT-INR	1.2 (1.1–1.2)	1.1 (1.0–1.2)	0.073
APTT (s)	42.0 (27.4–54.5)	34.2 (21.4–60.9)	0.055
ACT (%)	1 (12.5)	3 (8.3)	0.566
APT (%)	5 (62.5)	13 (36.1)	0.240
Multiple dosage (%)	0	2 (5.6)	1.000

Mean (SD) or median (range)

ACT, anticoagulation therapy; APTT, activated partial thromboplastin time; ATT, antithrombic therapy; BMI, body mass index; ASA-PS, American Society of Anesthesiologists-Physical Status; CCI, Charlson Comorbidity Index: TG18, Tokyo guideline 2018; WBC, white blood cell; Hb, hemoglobin, Plt, platelet; CRP, c-reactive protein; Alb, albumin, γGTP, gamma glutamyl transpeptidase; PT-INR, prothrombin time-international normalized ratio

**Table 4:** Multivariate analysis of preoperative risk factors for blood loss over 100 mL

	OR (95% CI)	*P* value
Alb (3.5≤)	0.207 (0.031–1.400)	0.106
PT-INR (1.5≤)	0.000 (0.000–infinity)	0.994
APTT (40≤)	7.380 (1.110–49.100)	0.039

OR, odds ratio; CI, confidence interval; Alb, albumin; PT-INR, prothrombin time-international normalized ratio; APTT, activated partial thromboplastin time

## Discussion

Previous studies showed that emergency LC for AC can be safely perform with patients taking ATT [11–14]. However, there were significant differences in patient characteristics in these studies. Elder population is a risk factor for bleeding when using ATT [18–21]. Also, liver disease, renal disease, and inflammation itself increases the bleeding risk [22–24]. From our study APTT was in independent risk factor for bleeding over 100 mL. PT and APTT are both valuable factors to measure the time it takes plasma to clot when various substances are added. When PT and APTT are both are prolonged, there is a problem in final common pathway of coagulation. This is observed in liver disease and disseminated intravascular coagulation. On the other hand, only APTT is prolonged when there is a problem in intrinsic pathway of coagulation. This is observed in several inherited bleeding disorders and due to several ACT such as heparin and direct oral anticoagulants. Therefore, patient characteristics need to be matched to exclude these affects. In this study, we showed that APTT affected the blood loss, while ACT and APT did not. Also, there was no significant difference in other postoperative complications between groups. These results suggest that ATT will not increase the risk of bleeding and emergency LC for AC is feasible and safe for patients taking ATT.

Prevention of bleeding by meticulous hemostasis during the laparoscopic surgery is a fundamental principle. Therefore, prompt management is required even when minor bleeding occurs. Recent improvement in surgical instruments have contributed to less bleeding. Argon beam coagulator, microwave coagulator, and ultrasonic coagulating shears have been developed and used to stop bleeding from the gallbladder bed during LC for AC [25]. Also, conjunction with mechanical compression using dry hemostatic agents help slower the bleeding. We believe that these new instruments have contributed to control the bleeding during surgery despite taking ATT in our study.

Multiple dosage of ATT is sometimes observed. Previous studies evaluated the risk of bleeding when taking multiple dosage of ATT; however, results differed between studies [26–30]. Xu et al. reported that age over 90 was a risk of higher bleeding when taking multiple dosage of ATT [28]. Kawamoto et al. reported that multiple dosage of ATT showed bleeding from the area of surgery and also gastrointestinal bleeding after surgery [2]. Multiple dosage was not a risk factor of bleeding from results of this study; however, we should still be aware of the risk of bleeding when elder is taking multiple dosage of ATT.

This study has several limitations. This is a retrospective study at a single institution, so the sample size, especially the number of patients taking ATT is small. Therefore, we could not completely match the patient characteristics and classify the ATT according to the types of antithrombotic agents that were administered. Also, not all cases with ATT had emergency LC, since TG18 also recommends biliary drainage as an alternate treatment for higher TG18 grade [31]. Therefore, the safety of emergency LC with those taking ATT with higher TG18 is still unclear. Further studies with a larger sample are necessary to clarify these limitations.

## Conclusion

Emergency ATT does not affect the blood loss or complications during emergency LC for AC. Controlling intraoperative bleeding is essential for a safe postoperative outcome.

## Data Availability

The data that support the findings of this study are available from the corresponding author upon reasonable request.

## Abbreviations

ATT: Antithrombic therapy
LC: Laparoscopic cholecystectomy
AC: Acute cholecystitis
TG18: Tokyo guideline 2018
ASA-PS: American Society of Anesthesiologists-Physical Status
CCI: Charlson Comorbidity Index
CRP: C-reactive protein
ACT: Anticoagulation therapy
APT: Antiplatelet therapy
PT-INR: Prothrombin time-international normalized ratio
APTT: Activated partial thromboplastin time
